# Measuring Laypeople’s Trust in Experts in a Digital Age: The Muenster Epistemic Trustworthiness Inventory (METI)

**DOI:** 10.1371/journal.pone.0139309

**Published:** 2015-10-16

**Authors:** Friederike Hendriks, Dorothe Kienhues, Rainer Bromme

**Affiliations:** Institute for Psychology, University of Muenster, Muenster, Nordrhein-Westfalen, Germany; Tilburg University, NETHERLANDS

## Abstract

Given their lack of background knowledge, laypeople require expert help when dealing with scientific information. To decide whose help is dependable, laypeople must judge an expert’s epistemic trustworthiness in terms of competence, adherence to scientific standards, and good intentions. Online, this may be difficult due to the often limited and sometimes unreliable source information available. To measure laypeople’s evaluations of experts (encountered online), we constructed an inventory to assess epistemic trustworthiness on the dimensions expertise, integrity, and benevolence. Exploratory (*n* = 237) and confirmatory factor analyses (*n* = 345) showed that the Muenster Epistemic Trustworthiness Inventory (METI) is composed of these three factors. A subsequent experimental study (*n* = 137) showed that all three dimensions of the METI are sensitive to variation in source characteristics. We propose using this inventory to measure assignments of epistemic trustworthiness, that is, all judgments laypeople make when deciding whether to place epistemic trust in–and defer to–an expert in order to solve a scientific informational problem that is beyond their understanding.

## Introduction

When gathering knowledge, people depend on the testimony of others [1,2]. Especially when it comes to scientific information, laypeople must defer to experts because they lack the relevant background knowledge [3–5]. It may well be hard for a layperson dealing with scientific information to assess “what is true” because direct observation of a scientific discovery (e.g., nanoparticles or genes) is often impossible and the veracity of scientific information about such discoveries cannot be judged firsthand [6], because fully comprehending this information would require specialized knowledge that only experts in the field possess. Hence, one solution is for laypeople to ask “whom to believe”. This means deferring to and relying on those experts who have deeper knowledge about a topic and may be able to provide information about the validity and veracity of information [5,7].

However, uncritical deference to others exposes one to the risk of being lied to or cheated [8,9]. This might happen accidentally, due to the informant’s false beliefs (e.g., an expert has acquired only preliminary results so far); or deliberately, due to selfish, and sometimes even malevolent intentions (e.g., an expert has commercial interests) [1]. Researchers have argued that epistemic trust involves not only believing others uncritically but also being vigilant regarding deception and misinformation [1,2,10]. In this article, we adopt this conception of epistemic trust in experts.

Because we wanted to investigate how people decide to trust an expert regarding scientific information, we developed an inventory measuring laypeople’s ascriptions of epistemic trustworthiness to an expert. We also wanted to determine the underlying dimensions of such epistemic trustworthiness. And, finally, we wanted our inventory to be applicable especially for trustworthiness ascriptions to experts who are communicating information online. Why this is important will be explicated in the following.

### A digital knowledge society

In our digital age, (scientific) information is accessible at all times. The Internet, smartphones, and tablet computers enable a large public to access a vast amount of information in just a few seconds. In 2014, more than 60% of Internet users usually turned to the Internet to get information on scientific topics [11], and the percentage is even higher among young users [12,13]. Online, one can find countless experts who share knowledge about their area of expertise and even their recent research findings in a number of ways. Many experts are questioned or cited by science journalists [14], and an increasing number of scientists entertain or contribute to science blogs [15,16]. Furthermore, already in 2011, a total of 11% of the world’s scientific articles were published open access [17].

Such developments pose two challenges for laypeople. First, they have to decide who is a trustworthy expert without themselves possessing much topic-specific knowledge. This might be difficult because there is no face-to-face interaction and mostly not even a visual representation of the speaker. Therefore, the usual credibility cues (facial expressions, gestures, appearance) do not apply. Also, valid cues for determining the credibility of a source of information (e.g., area of expertise, affiliation, education) might not be salient or even be hidden in the site notice [18]. As a result, a layperson may well use all the information provided, most importantly the text itself, to infer a source’s trustworthiness. Besides the content, the spelling and syntax of a text or the use of technical language may serve as valuable cues for determining the expertise and intentions of its source.

Second, there is an even greater risk of being misinformed online than in the traditional news media. Because virtually anyone may publish online without gatekeepers such as publishers or editors, it is up to the recipient to assess online sources for trustworthiness and information on their credibility [19,20]. For example, it might be hard to differentiate advertisements from true information or cheaters from reliable informants. Moreover, from developmental psychology we know that young children accept information from testimony readily, even if it is in conflict with their own beliefs [21, 22]. Furthermore, early readers prefer written information over information that is presented by spoken testimony [23, 24]. Thus, being mostly text-based and sometimes presented to be authored by a reliable (but in fact, a possibly deceptive) source, online science-based information might evoke a similar trust bias in recipients, especially because they only possess a bounded understanding of the underlying science [4]. In the studies at hand, we aim to show which inferences about the trustworthiness of sources of online science-based information are made by recipients, guiding their reasoning about the information’s believability. Such trustworthiness inferences may help laypeople to overcome the challenges that lie within the reception of online science-based information.

### What is credibility and what is epistemic trustworthiness?

What are trustworthiness judgments based on? Classic research on credibility has suggested a variety of qualities. Aristotle defined three major character properties that a speaker must possess in order to be persuasive: “(i) practical intelligence […], (ii) a virtuous character, and (iii) good will” [25]. In the 1950s, within a framework of growing research on persuasion, the Yale Group introduced the idea of communicator credibility, which was defined as consisting of expertness and trustworthiness [26]. Over the years, several scales have been developed to measure source credibility, most often using pairs of antonymous adjectives to form scales rating the credibility of a speaker [27,28]. These have included various dimensions such as competence, safety, dynamism, attractiveness, or composure, to name just a few [28]. McCroskey and Teven [29] developed an inventory (items were pairs of antonymous adjectives) to measure the credibility of well-known sources that included competence, good will, and trustworthiness.

By definition, the term “source credibility” describes positive characteristics that will lead to the acceptance of a message [28]. Trust, on the other hand, is defined by a dependence of a trusting actor on the trusted person or entity [30] combined with a vulnerability to risk [31]. Accordingly, “epistemic trustworthiness” describes those features of experts that decide whether recipients will depend on and defer to them due to their own limited resources [7,32,33]. Trust research has extensively studied which features of a trustee are valued as trustworthy in various contexts. Interestingly, the identified dimensions were quite similar to those found in credibility research. In interpersonal trust relationships between, for example, supervisor and employee, influential work by Mayer, Davis, and Schoorman [31] has identified expertise, integrity, and benevolence as crucial factors of trustworthiness. Research on epistemic trust in developmental psychology has shown in detail that children use a source’s presumed knowledge, honesty, and intent to decide which source to believe and learn from [2,34–36]. When adults decide which expert they can rely on to acquire knowledge (who is then considered the epistemic authority [37]), expertness and objectivity seem to be the most important indicators [38]. Furthermore, qualitative data show that laypeople spontaneously report the following features of a scientific expert when asked to make trust evaluations: expertise, objectivity or work ethic, and moreover, potential interests that stand in conflict with the public interest [39]. Both empirical and theoretical research indicates that trust in scientists should be based on not only their epistemic quality but also their moral integrity [40,41] and the application of their work for the benefit of society [42].

We can conclude that three dimensions emerge in empirical and theoretical work on credibility and trustworthiness: expertise (also called competence or ability); integrity, which may include all facets related to the source’s honesty, objectivity, and adherence to recognized standards, and, as some have added, benevolence, which summarizes the intentions or the good will of an expert. Thus, we assume that a scientist who wants to be considered epistemically trustworthy should be knowledgeable, act with (scientific) integrity, and practice benevolence toward others.

### Present studies

We aimed to develop a new inventory for measuring such epistemic trustworthiness judgments for two reasons: First, the scales formulated for measuring source credibility focus on source characteristics that deliberately refer to the persuasive quality of the source. However, when assessing the *epistemic* trustworthiness of sources, recipients aim not to be easily persuaded or misinformed. Due to this important theoretical distinction between credibility and epistemic trustworthiness, we propose the creation of a new inventory. Second, the available measures of credibility mostly have been designed to evaluate a known source or speaker. Nowadays, in many (online) settings, information about the source is often scarce. Hence, items should be fit to measure trustworthiness assessment that is inferred from very little information about the source such as only text features. Our aim is to create an inventory that is able to measure ascriptions of epistemic trustworthiness in an online context characterized by limited source information.

Drawing on theory and empirical research, we propose that epistemic trustworthiness should be conceptualized as consisting of the following dimensions: the expert’s a) expertise and knowledge about the topic of interest, b) reliability and integrity in adhering to scientific standards, and c) benevolence toward others or even society. When developing the inventory, we took into account previous work on scales measuring credibility [27,29], but adapted this to fit the theoretical ideas underlying the concept of epistemic trustworthiness. Furthermore, to the best of our knowledge, these credibility scales have not yet been either transferred to an online context or validated with both exploratory and confirmatory factor analysis.

Our inventory consisted of oppositional adjectives (antonyms) rated on a 7-point scale like a semantic differential. In a first study, we explored the factor structure of this inventory, which yielded a three-factor structure. These factors were labeled ‘expertise’, ‘integrity’, and ‘benevolence’. In a second study, we aimed to check the fit of the model derived from Study 1 with a new data set. Because the new data matched the derived model, we succeeded in developing an inventory with three subscales for measuring epistemic trust in unfamiliar experts: the *Muenster Epistemic Trustworthiness Inventory* (METI). In a third study, we experimentally varied source characteristics to show that participants rate a source using the three scales of the METI in a differentiated way.

## Study 1

### Materials and methods

#### Development of items

Participants received 18 word pairs. These were constructed to be exact semantic opposites (i.e., antonyms). Some words were derived from theory; others were taken from existing inventories measuring source credibility [27,29]. Words were included after 12 colleagues conducting trust research judged them to have a clear relation to the concept of epistemic trust. In the resulting inventory, participants were asked to rate the scientific expert on Likert scales ranging from 1 (e.g., *professional*) to 7 (e.g., *unprofessional*).

#### Procedure

Using an online survey software, we constructed an online survey. Participants who had previously signed up to be invited to studies carried out by our research group were informed about the study via e-mail. Participants could choose the time and location of their participation. Participation was voluntary. Participants who answered all items could take part in a lottery to win one of 20 vouchers from a well-known online store (worth €10 each).

After a set of demographic questions, participants read a fictitious science blog entry written purportedly by an expert blogger (see S1 Appendix). This expert blogger described his own research, explaining that he had conducted an experimental study to test whether a (fictitious) neuroenhancing medication would enhance neurocognitive functioning. The blog entry was embedded in a science blog of a fictional scientific institute to which the expert blogger was affiliated. In a side panel, a box included some source information, namely, the name of the expert blogger, a blurred picture of him (so that participants would not judge his attractiveness), and the information that he belonged to a research group at an institution (which sounded reputable but was actually fictitious). The blog was designed as if it were a blog reporting research conducted at this institution. After participants read the science blog, we surveyed the epistemic trustworthiness they ascribed to the expert blogger with the previously described inventory. Finally, we surveyed strategies that participants would use gathering more information, but this data is not relevant for the results of this study.

#### Sample

Of 300 people who had decided to click the link to open the survey, a total of *n* = 237 participants completed the survey. They were all included in the analyses. Due to the settings of the survey software, there were no missing data. Participants in the final sample were between 19 and 47 years of age (*M* = 25.39, *SD* = 4.38) and predominantly female (75.5%). The majority reported having graduated from the academic upper secondary school track (i.e., with a German *Abitur*, *n* = 116). A further *n* = 110 participants reported having a university degree.

#### Ethics statement

All participants had consented to be contacted via e-mail prior to this study. We contacted each potential participant only once via e-mail and gave them a link to the online survey. Upon accessing the survey, participants were informed that participation was voluntary, filling out the survey would take about 10 minutes, and their data would be stored anonymously and used solely for research purposes. All participants took part on a voluntary basis at the time and location of their free choosing. At the end of the study, participants were informed that all materials used (i.e. the blog entry) were made up for the purpose of the study and that all persons mentioned as well as the drug mentioned were fictitious.

All data were collected anonymously. When personal data (e.g., e-mail addresses) were obtained, these were stored separately from the survey data and deleted upon sending out one voucher to each of the lottery winners. The study was conducted in full accordance with the Declaration of Helsinki, and the German Psychological Society’s (DGPs) ethical guidelines [43] (based on the APA guidelines [44]). Here, it is outlined that those psychological studies do not require the explicit consent of participants based on the full disclosure of study means and goals that do not expose participants to physical, emotional or financial stress beyond every day experience. Because this was the case in our research and also, because participants were informed of voluntary participation that could be stopped at any time, and because all data were analyzed anonymously we did not consult an institutional review board.

All raw data will be retained for at least five years after publication and will be made available to professionals for confirmation of analyses and results [44].

### Results

#### Appropriateness of the data

Sample size was adequate for exploratory factor analysis, because the Kaiser–Meyer–Olkin (KMO) value of .92 corresponds to a “superb” sample size [45]. For individual items, all KMO values were > .88 and well above the acceptable limit of .5 [45]. No items had to be excluded due to item intercorrelations greater than .9 [45]. Bartlett’s test was highly significant, *χ* ^2^(120) = 2491.82, *p* = .000. Hence, we could assume that the data were appropriate for factor analysis because all correlations between variables differed from zero [45].

#### Decisions on method and number of factors retained

All analyses were computed using SPSS 22 statistical analysis software. We decided to use the maximum likelihood technique. This technique is optimal if data are approximately normally distributed [46], which we assessed by inspecting the probability–probability plots (P–P plots). We judged direct oblimin rotation to be adequate for the data, because intercorrelations between the factors were reasonable. Using the scree-plot criterion, we assumed a factor solution with three factors, which was also concurrent with theoretical assumptions. Furthermore, as Costello and Osborne [46] suggest, exploratory factor analyses with two, three, and four factors were computed manually to find the “cleanest” factor structure (without double loadings). Again, the three-factor solution was accepted because after exclusion of one item, no double loadings were found and factors were easy to interpret, whereas in a two-factor solution two items (*honest–dishonest* and *helpful–unhelpful*) loaded highly on both factors and in a four-factor solution, factors with two items only were found. Therefore, the three-factor solution, which did not produce double loadings, but three factors with four items at least, proved to explain the data best. The three factors explained 61.66% of the total variance.

#### Factor solution

One item (“*reliable–unreliable”*) was excluded due to a significant double loading >.32 on two factors, and a second item (“*impartial–partial”*) was discarded for not loading significantly at all [46]. As a result, the final factor solution consisted of 16 items (see Table 1). One factor consisted of 7 items all relating to the epistemic quality and competence of the expert blogger. Thus, this factor was labeled *‘expertise’*. A second factor consisted of 5 items relating to the expert blogger’s adherence to scientific standards, with regard to being fair and honest. This factor was labeled *‘integrity’*. A third factor consisted of 4 items related to the expert blogger’s concern for other people and society. Hence, this factor was labeled *‘benevolence’*. The internal consistencies of all three factors were very good. The values for Cronbach’s alpha were: α = .91 for expertise, α = .82 for integrity, and α = .90 for benevolence.

**Table 1:** Pattern matrix for the three factor solution.

Item	Expertise	Integrity	Benevolence
competent–incompetent	.806		
intelligent–unintelligent	.704		
well educated–poorly educated	.806		
professional–unprofessional	.712		
experienced–inexperienced	.808		
qualified–unqualified	.825		
helpful–hindering	.367		
sincere–insincere		.754	
honest–dishonest		.841	
just–unjust		.572	
unselfish–selfish		.347	
fair–unfair		.422	
moral–immoral			.907
ethical–unethical			.919
responsible–irresponsible			.741
considerate–inconsiderate			.527

Internal consistency of the factors: Expertise, α = 0.908 (7 items), Integrity, α = 0.821 (5 items); Benevolence, α = 0.904 (4 items)

#### Correlations between factors

We examined correlations between factors (see Table 2). The factor expertise correlated with integrity (*r* = .51) and with benevolence (*r* = .37). The highest correlation was found between integrity and benevolence (*r* = .63). We computed partial correlations between the three factors to see how far they overlapped. Expertise correlated with integrity at *r* = .35, controlling for benevolence. Integrity correlated with benevolence (without the influence of expertise) at *r* = .58. Expertise and benevolence correlated at *r* = .16 (without integrity).

**Table 2:** Factor correlation matrix for the three factors derived from EFA.

Factor	Expertise	Integrity	Benevolence
Expertise	1.00	0.51	0.37
Integrity		1.00	0.63
Benevolence			1.00

### Interim Discussion of Study 1

In this first study, we implemented a measurement of epistemic trustworthiness that we have decided to call the *Muenster Epistemic Trustworthiness Inventory* (METI) in the following. A three-factor solution was obtained with the factors expertise, integrity, and benevolence. Correlations between the three factors did not lead us to question the acceptability of the model [45]. On the contrary, the three-factor solution represented three distinctive dimensions. Of course, as all three factors describe the characteristics for choosing a person as a trustworthy source of knowledge, factors are related and show medium to high correlations.

Unexpectedly, the item “*helpful–unhelpful*” loaded on the factor expertise. Due to the single loading on this factor, we included this item in the inventory (with the expectation that if the item was related to any other factor or had to be excluded from the inventory, this would be revealed in the subsequent confirmatory factor analysis). Similarly, the item “*unselfish–selfish*” loaded unexpectedly on the factor integrity, although the loading was not very high. Again, we included the item in the inventory to further examine its reliability through CFA.

In sum, in the first study, we achieved our objective of identifying three different dimensions of epistemic trustworthiness that can be labeled ‘expertise’, ‘integrity’, and ‘benevolence’. Furthermore, we established that when participants evaluated an expert who had been encountered online for the first time and on whom very scarce information was accessible, the METI was applicable for measurement.

## Study 2

In Study 2, we aimed to test the three-factor structure of our instrument, the METI, by assessing data from a new sample with confirmatory factor analysis. This should provide further evidence supporting the conclusion derived from Study 1 that epistemic trustworthiness ascribed to unfamiliar experts by laypeople is indeed composed of the three subsidiary dimensions expertise, integrity, and benevolence.

### Materials and Methods

#### Procedure

We conducted an online survey using an online survey software Participants were students who were approached while attending lectures at a German university and asked to leave their e-mail address for a later invitation to participate in the study. They then received a single e-mail inviting them to take part in the study online; participation was voluntary. Participants who answered all items could take part in a lottery and win one of 20 vouchers from a well-known online store (worth €10 each).

The study materials and procedure were exactly the same as in Study 1. Hence, the same ethical considerations apply to Study 2 as well.

#### Sample

Of 406 people who activated the online survey, a total of *n* = 345 participants completed it. Only those who completed the survey were included in analyses. Participants were *M* = 21.77, *SD* = 3.93 years of age and 69.3% of the sample were female. Regarding their education, the majority of participants (84.9%) reported having graduated from upper secondary school (i.e., having a German *Abitur*, *n* = 293). A further 42 participants reported having a university degree. There were no missing data due to settings of the survey software.

#### Measure for epistemic trustworthiness

Based on the results from Study 1, the Muenster Epistemic Trustworthiness Inventory (METI) was reduced to 16 items: 7 items for the factor expertise, 5 items for the factor integrity, and 4 items for the factor benevolence.

### Results

#### Appropriateness of data

The sample size was sufficient for conducting confirmatory factor analysis (47). Regarding r > .85 as the critical criterion, no item had to be excluded due to high item intercorrelations [43]. Furthermore, a normal distribution could be assumed from visually inspecting histograms and P–P plots. Values of skewness and kurtosis are attached in S1 Table. All values for skewness/kurtosis were distinctly below 2.0/7.0, so that conducting CFA was permissible [48]. Nonetheless, Mardia’s test was significant (multivariate normality = 42.51, critical ratio = 18.66).

#### Model estimation

Analyses were computed using the software program SPSS AMOS (Version 22). The three dimensions of the METI (expertise, integrity, and benevolence) were indicated latent variables predicted by their irrespective items as indicators. We chose the maximum-likelihood method for model estimation, because the data were normally distributed [47]. Because Mardia’s test proved significant, we performed a Bollen–Stine bootstrap [47]. For each latent variable, the loading of the most reliable indicator was fixed to 1 [47]. Two items were excluded from the model (“*helpful–hindering”* from the factor expertise and “*unselfish–selfish*” from integrity), because they each had a low communality and additionally were not unambiguously associated only with their respective constructs. Post hoc modifications were indicated by analysis, comprising a better fit. Hence, one covariance between errors was allowed; those errors were all related to indicators of the latent variable Integrity (namely *“fair–unfair”* and *“just–unjust”*).

#### Model fit

Each factor loading was highly significant at the *p* = .001 level. Thus, local model fit can be assumed (see Table 3). To evaluate the model fit, we used directives given by Schreiber and colleagues [49]. Furthermore, we followed recommendations by Beauducel and Wittmann [50] for reporting model fit indices.

The chi-square test proved significant, which is usual for a sample size lower than 300 [47], *χ* ^*2*^ = 165.78, *df* = 73, *p* = .000. Nonetheless, the ratio of *χ* ^*2*^ to degrees of freedom was sufficient, *χ* ^*2*^ */df* = 2.271. Compared to the independence model, the following fit indices were obtained: The comparative fit index was CFI = .97, above the acceptance criterion of > .95; the Tucker–Lewis index was TLI = .96, again above the acceptance criterion (> .95). The root mean square error of approximation was RMSEA = .06. Values < .08 indicate a good fit. The standardized root mean error residual was SRMR = .06 and below the acceptance criterion (≤ .08). Thus, all values indicated a good fit between the model and the observed data. Standardized parameter and unstandardized parameter estimates are reported in Table 3.

**Table 3:** Standardized and unstandardized coefficients following CFA.

Observed variable	Latent Construct	β	*B*	*SE*
competent–incompetent	Expertise	0.866	0.953	0.043
intelligent–unintelligent	Expertise	0.684	0.749	0.051
well educated–poorly educated	Expertise	0.825	0.890	0.044
professional–unprofessional	Expertise	0.748	0.916	0.054
experienced–inexperienced	Expertise	0.719	0.781	0.049
qualified–unqualified	Expertise	0.866	1	
sincere–insincere	Integrity	0.826	1	
honest–dishonest	Integrity	0.788	0.948	0.066
just–unjust	Integrity	0.551	0.550	0.056
fair–unfair	Integrity	0.572	0.581	0.057
moral–immoral	Benevolence	0.826	1	
ethical–unethical	Benevolence	0.764	0.886	0.058
responsible–irresponsible	Benevolence	0.736	0.921	0.064
considerate–inconsiderate	Benevolence	0.789	0.927	0.056

All standardized regression weights were significant, p < .001.

#### Correlations between factors

We computed correlations between factors (see Table 4). The factor expertise correlated with integrity at *r* = .63 and with benevolence at *r* = .52. The highest correlation was found between integrity and benevolence, *r* = .71. Again, we computed partial correlations. Expertise and integrity correlated at *r* = .39 (without benevolence), integrity and benevolence at *r* = .51 (excluding expertise), and benevolence and expertise correlated at *r* = .18 (without the influence of integrity).

**Table 4:** Factor correlation matrix.

Factor	Expertise	Integrity	Benevolence
Expertise	1.00	0.63	0.52
Integrity		1.00	0.71
Benevolence			1.00

### Interim Discussion of Study 2

In Study 2, we successfully fitted the theoretical three-factor model to a new data set. This replicated the three-factor structure of our inventory. The elimination of two additional items (“*helpful–unhelpful”* and *“unselfish–selfish”*) resulted in a final version of the METI with 14 items. Correlations between factors were again assessed, but, as argued before, we considered these to be unproblematic because all three factors are related to a comprehensive theoretical construct [45], namely epistemic trustworthiness.

For integrity, communalities of the items “*just–unjust”* and “*fair–unfair”* were quite low. This is possibly due to a correlation of error terms indicated by the analysis. This correlation might be explained by very close semantical relation. It may also imply that integrity is a heterogeneous factor [47]. In fact, this factor seems to comprise items that relate to the good character of an expert (e.g., *honest*) as well as items that address an expert’s values (e.g., *just*, *fair*). By implication, integrity includes both an expert’s sincerity and appropriate values.

## Study 3

In Study 3, we aimed to show that participants when evaluating sources use the three subscales of the METI in accordance with theoretical ideas and a differentiated way. For this purpose, we constructed six short descriptions of expert sources, which epistemic trustworthiness (on the METI) was to be rated by participants. In these descriptions, it was varied, if a source was of high versus low expertise, integrity or benevolence respectively.

### Design of the experiment

A short blog entry about a study from the domain neurology was constructed (see S2 Appendix). This blog entry was followed by six short descriptions of six fictitious persons, who were indicated as potential authors of the blog entry. These author descriptions were constructed in such a way that they entailed either a very trustworthy or a non-trustworthy description for each dimension of epistemic trustworthiness (expertise, integrity and benevolence). This was done by describing exemplary characteristics of informants, in consistence with theoretical ideas about each epistemic trustworthiness dimension’s purport (See Table 5 for materials). All authors were indicated to be pertinent for the scientific domain neurology. Participants were asked to rate the trustworthiness (on the METI) for all six authors, which were introduced in a randomized order.

**Table 5:** Study 3 –Materials: Descriptions of potential authors (translation from German).

		Independent variable A: Epistemic trustworthiness dimension targeted in description of the source		
		Expertise	Integrity	Benevolence
**Independent variable B: Description entailing low/high epistemic trust-worthiness**	**Low**	[Name] has been studying medicine for three semesters. On the side, he works in gastronomy. In the context of his studies, he takes classes in anatomy, physiology and neurology.	Dr. [Name]: Researching neurologist at a university. In 2012, colleagues reported that he had withheld findings, which were contradicting his previous results. Those retained findings would have called some of his previous studies into question.	Dr. [Name]: Researching neurologist at a university. He was accused to have released a drug for his company, without adequately researching adverse effects first. His main concern was said to be making profit.
	**High**	Prof. Dr. [Name]: Professor in neurology at a university and internationally recognized expert for the neurology of migraine patients. For several years, his research has been directed at sleep- and concentration-disorders.	Dr. [Name]: Researching neurologist at a university. He is an active member in the initiative „Open Science“. In this initiative, researchers pledge to publish all their studies’ materials and data so that they may be verified by other, impartial experts.	Dr. [Name]: Researching neurologist at a university. He is championing of science producing insights that prove advantageous for the whole society. His goal is that his research contributes to neurology helping patients.

ET = Epistemic Trustworthiness; all descriptions were translated from German by authors

### Hypotheses

The aim of study 3 was to assess if the METI‘s three subscales are used in a differentiated way for recognition of extraordinary or insufficient expertise, integrity and benevolence. In accordance, we expected that if a description of an author high in expertise, integrity or benevolence was given, participants would give high ratings on the METI’s relevant subscale, and that the opposite would be true for descriptions of low expertise, integrity or benevolence. However, in studies 1 and 2, moderate to high correlations between factors were found. For this reason, we did not expect total independence of the subscales. Thus, we expected:

H_1_: The margin of ratings on the METI subscale ‘expertise’ should be largest between the two authors with descriptions of low and high expertise, in contrast to the margin between ratings of ‘expertise’ comparing the two authors with descriptions of low and high integrity or comparing the two authors with descriptions of low and high benevolence.

H_2_: The margin of ratings on the METI subscale ‘integrity’ should be largest between the two authors with descriptions of low and high integrity, in contrast to the margin between ratings of ‘integrity’ comparing the two authors with descriptions of low and high expertise or comparing the two authors with descriptions of low and high benevolence.

H_3_: The margin of ratings on the METI subscale ‘benevolence’ should be largest between the two authors with descriptions of low and high benevolence, in contrast to the margin between ratings of ‘benevolence’ comparing the two authors with descriptions of low and high expertise or comparing the two authors with descriptions of low and high integrity.

### Methods

#### Procedure

We conducted an online survey using an online survey software Participants were invited only once via email (using previously collected email addresses for the purpose of inquiry for further studies). Furthermore, advertisements were posted on the website of the students’ general committee of a large German university and of a magazine directed at laypeople interested in psychology. Participants who answered all items could take part in a lottery and win one of 10 vouchers from a well-known online store (worth €10 each).

The procedure was exactly the same as in Study 1 and 2. Hence, the same ethical considerations apply to Study 3 as well.

#### Sample

243 people activated the online survey and 152 people completed it. Upon inspecting the data, we found large variation in completion time (between 2.8 minutes and 2600.3 minutes). Hence, regarding completion time, we dropped the top and the bottom 5% of participants, including only those with a completion time between 5.20 min and 42.54 min resulting in a sample of *n* = 137. Those participants were *M* = 26.22, *SD* = 5.24 years of age and 75.2% of the sample were female. Regarding their education, the majority of participants (65.0%) reported having graduated from college or university. 31.4% had graduated from upper secondary school (i.e., having a German *Abitur*). A further five participants reported a level of different qualification (no degree, secondary school, in-firm training). There were no missing data due to settings of the survey software.

#### Measure for epistemic trustworthiness

Based on the results from Study 2, the Muenster Epistemic Trustworthiness Inventory (METI) was reduced to 14 items: 6 items in the subscale ‘expertise’, 4 items in the scale ‘integrity’, and 4 items in the scale measuring ‘benevolence’.

### Results

#### Appropriateness of data

Using GPower [51], prior to data collection it was determined that at least 62 participants would be needed for detecting a medium sized effect, η_p_^2^ = .05, with power (1—β) set at 0.95, the correlation among repeated measures set at *r* = .00 and α = .05 (two-tailed).

For all dependent variables, Kolmogorov-Smirnov-Tests were conducted to assess the normal distribution of the data. For all dependent variables (measured for each condition), normality cannot be assumed. However, ANOVA is robust to deviations from normality, if due to skewness [52]. Furthermore, ANOVA is conservative in the case of positive excess kurtosis, which was mostly the case in our data (see S2 Table for skewness and kurtosis), and more liberal in the case of negative excess kurtosis [52]. Furthermore, in repeated measurements, significant results are found easily due to correlations among measurements. Due to this, we focus on the interpretation of effect sizes.

We conducted a 3x2 repeated-measures ANOVA in SPSS 22 for all three dependent variables (ratings on the METI subscales ‘expertise’, ‘integrity’ and ‘benevolence’) for determining the main effects of dimension of epistemic trustworthiness (description targeted at expertise, integrity or benevolence) and manifestation of epistemic trustworthiness (description entailing low or high trustworthiness) and the interaction effect between the two independent variables on each of the subscales of the METI separately.

To determine how the margin of trustworthiness ratings (on each of the METI’s dimensions) between authors being described on the same dimension of epistemic trustworthiness (ET), but with different manifestation, would compare to the margin between authors with different manifestation of ET on another dimension of ET, we conducted planned contrasts [53] and report the effect size *r* [45]. Greenhouse-Geisser corrected F-values are reported if sphericity of variances cannot be assumed.

**Fig 1:**
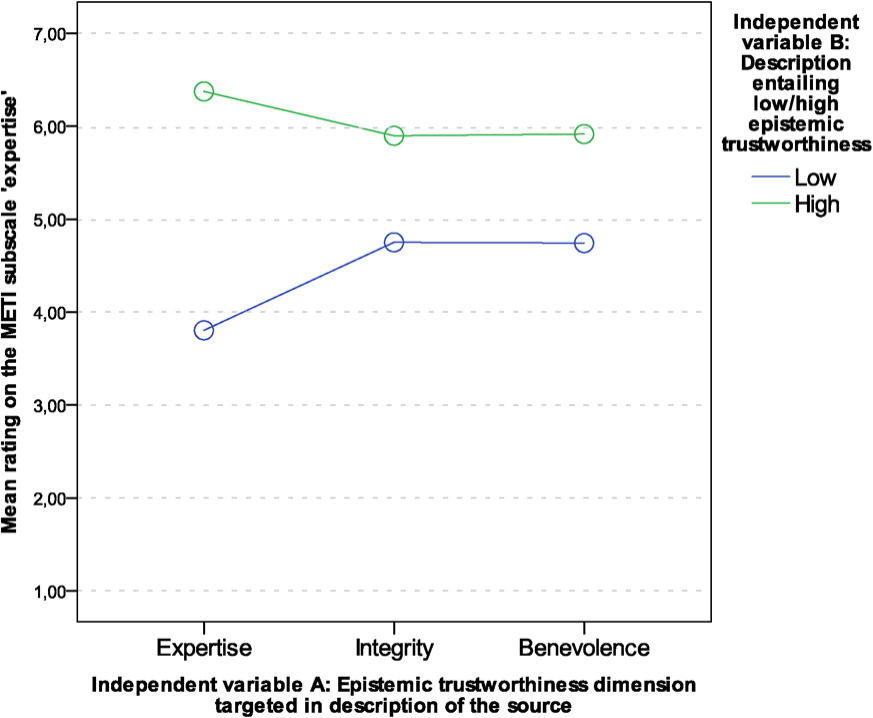
Interaction graph: Mean ratings on the scale ‘expertise’.

For ‘expertise’, all main effects and the interaction were significant with *p* < .001 (main effect of dimensions of ET: *F* (1.91, 259.99) = 10.69, *p* < .001, η_p_^2^ = .07; main effect of manifestation of ET: *F* (1, 136) = 590.25, *p* < .001, η_p_^2^ = .81; interaction of dimensions and manifestation of ET: *F* (1.76, 240.01) = 100.62, *p* < .001, η_p_^2^ = .43. Simple planned contrasts were conducted to break down the interaction. Comparing the margin in ratings between authors who were described to be of low or high expertise, there was a significant difference to the margin between authors described to be low or high in integrity (*F* (1, 136) = 146.59, *p* < .001, *r* = .72), and benevolence (*F* (1, 136) = 114.92, *p* < .001, *r* = .68). The interaction graph in Fig 1 shows that the margin is larger, when descriptions were targeting expertise than when they were targeting integrity or benevolence. For means and standard deviations see Table 6.

**Table 6:** Means and standard deviations.

	Dependent Variable		
Condition (Author Description)	Expertise: Mean (SD)	Integrity: Mean (SD)	Benevolence: Mean (SD)
Expertise: Low	3.80 (.91)	4.62 (.84)	4.45 (.82)
Expertise: High	6.37 (.88)	4.99 (1.01)	5.01 (1.07)
Integrity: Low	4.75 (1.02)	2.40 (.92)	2.51 (1.00)
Integrity: High	5.90 (.90)	5.93 (.98)	5.79 (1.01)
Benevolence: Low	4.74 (1.08)	2.41 (1.04)	1.88 (.99)
Benevolence: High	5.91 (.88)	5.67 (.99)	5.87 (1.02)

**Fig 2:**
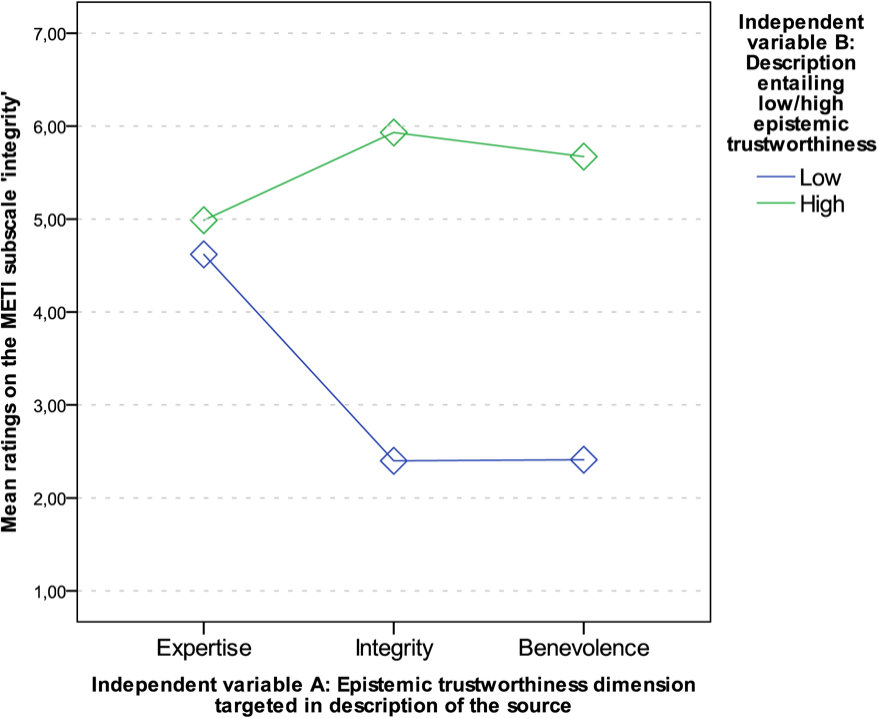
Interaction graph: Mean ratings on the scale ‘integrity’.

For ‘integrity’, all main effects and the interaction were significant with *p* < .001 (main effect of dimensions of ET: *F* (2, 272) = 78.89, *p* < .001, *r* = .37; main effect of manifestation of ET: *F* (1, 136) = 820.42, *p* < .001, η_p_^2^ = .86; interaction of dimensions and manifestation of ET: *F* (2, 272) = 341.28, *p* < .001, η_p_^2^ = .72. Planned contrasts were conducted to break down the interaction. Comparing the margin in ratings between authors who were described to be of low or high integrity, there was a significant difference to the margin between authors described to be low or high in expertise (*F* (1, 136) = 490.69, *p* < .001, *r* = .88), and benevolence (*F* (1, 136) = 4.65, *p* < .05, *r* = .18). Fig 2 shows that the margin between means in ratings of ‘integrity’ is larger, when descriptions were targeting integrity than when they were targeting expertise or benevolence (see Table 6 for means also).

**Fig 3:**
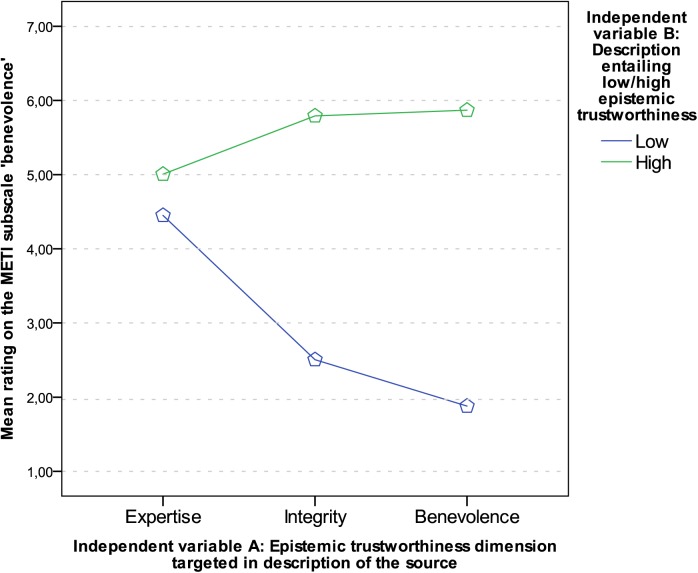
Interaction graph: Mean ratings on the scale ‘benevolence’.

For ‘benevolence’, all main effects and the interaction were significant with *p* < .001 (main effect of dimensions of ET: *F* (1.91, 259.99) = 79.34, *p* < .001, η_p_^2^ = .37; main effect of manifestation of ET: *F* (1, 136) = 994.58, *p* < .001, η_p_^2^ = .88; interaction of dimensions and manifestation of ET: *F* (2, 272) = 341.55, *p* < .001, η_p_^2^ = .72. Planned contrasts were conducted to break down the interaction. Comparing the margin in ratings between authors who were described to be of low or high benevolence, there was a significant difference to the margin between authors described to be low or high in expertise (*F* (1, 136) = 585.57, *p* < .001, *r* = .90), and integrity (*F* (1, 136) = 28.40, *p* < .001, *r* = .42). In Fig 3 it can be seen that the margin between means in ratings of ‘benevolence’ is larger, when descriptions were targeting benevolence than when they were targeting expertise or integrity (means and standard deviations can be found in Table 6).

### Interim Discussion of Study 3

In Study 3, it could be shown that the METI’s three subscales, expertise, integrity and benevolence are used in a differentiated way when a sources’ trustworthiness is rated, according to the description entailing either insufficient or extraordinary expertise, integrity or benevolence. Confirming hypothesis H_1_, the margin between ratings of expertise regarding authors described to be low versus high in expertise, was greater than the margin between authors described to be low versus high in integrity or benevolence. Both detected differences were large effects. Furthermore, confirming hypothesis H_2_, the margin between ratings of integrity regarding authors described to be low versus high in integrity was greater than between authors described to be low versus high in expertise (large effect) or benevolence (small effect). Lastly, hypothesis H_3_ was confirmed as well. The margin between ratings on the subscale benevolence regarding authors described to be low versus high in benevolence was greater than the margin between authors described to be low versus high in expertise (large effect) or integrity (moderate effect).

The results provide further evidence for the conclusion that the METI is able to measure epistemic trustworthiness in a differentiated way, breaking it down to the three dimensions expertise, integrity and benevolence. Again, it was confirmed that the scales are to some extent interrelated. This becomes evident fact that the margin between low and high descriptions of one trustworthiness dimension could be detected in all subscales of the METI (main effect of manifestation of ET). Furthermore, the interrelation between integrity and benevolence can be found again in smaller effect sizes of contrasts testing H_2_ and H_3_ comparing ratings on the scales integrity and benevolence to those comparing ratings on the scales integrity or benevolence separately to ratings on the scale expertise.

The fact that the dimensions integrity and benevolence are to some extent interrelated in this study may be due to the descriptions targeting only one of the three dimensions. One could easily imagine that a source described to be integer, but not benevolent (e.g. very honest and obedient to the rules of scientific conduct, but working only to be recognized by other experts and not to help others) would be rated very differently on the scales integrity and benevolence. In the study at hand, all authors were described to possess knowledge pertinent to the topic (neurology). In order to reduce redundancy for participants, descriptions of sources included only information on the dimension of epistemic trustworthiness targeted. Even though this was the case, highest differences in ratings of integrity were found between the two conditions, in which this dimension was targeted in the author description (high and low integrity). The same was true for ratings of benevolence. This is further evidence for a separability of the three dimensions.

Furthermore, results showed that recipients reduced ratings of trustworthiness on all dimensions, if an author was presented to be untrustworthy (on either of the dimensions of epistemic trustworthiness). These results show that recipients become epistemically vigilant toward a source’s trustworthiness [1], if they learn about the diminished expertise, integrity or benevolence of a source of scientific information.

To sum up, from examining results of study 3 it can be concluded that the three subscales, expertise, integrity, and benevolence are three separate dimensions of epistemic trustworthiness.

## Conclusions

The Muenster Epistemic Trustworthiness Inventory (METI) is composed of three subscales of epistemic trustworthiness that can be applied when researching laypeople’s judgments of epistemic trustworthiness in (online) science communication. It consists of 14 items.

The first subscale was labeled ‘expertise’ and contains six items. It reflects perceptions of the expert as truly knowledgeable, intelligent and highly trained in her or his domain. It relates to scales found in previous inventories in credibility and trustworthiness research labelled “qualification” [27], “competence” [29], or “ability” [31]. In the past, this dimension of epistemic trustworthiness has been found to be important. For example, previous accuracy of informants and knowledgeability of informants are strong determinants of children’s epistemic trust [2,36]. However, the expertise of a source has also been consistently reported to play a role in trust judgments in the context of health communication and online science communication [39,54,55].

The second subscale, ‘integrity’, consists of four items related to the expert’s good character and values, for example, being fair and honest. It reflects participants’ impressions of the scientist acting in line with scientific principles and within the norms of science. For scientists who communicate their findings to the public but also to the scientific community, this dimension is represented through characteristics such as sincerity and fairness. However, integrity, being defined as adherence to the norms and standards of one’s profession, may result in different measurement items depending on the context. In developmental psychology, we find best evidence supporting the importance of honesty for the constitution of epistemic trust [9,56]. Previous scales measuring credibility, never included integrity as one dimension, although some scales have contained similar items compared to those in METI’s Integrity scale, for example, “*honest–dishonest”* [28,29,57]. Cummings [39] describes the importance of a scientist’s integrity for trust judgments in science and health communication. Because expertise may be hard to determine without domain-relevant background knowledge, laypeople need more criteria than expertise alone to assess the epistemic authority of an expert they encounter. Laypeople seem to use evidence of an expert being motivated by scientific ideals such as objectivity to decide whether they regard her or him as trustworthy.

The third subscale, ‘benevolence’, contains four items relating to the expert’s orientation toward others or society, for example, her or his sense of responsibility and morality. This factor represents participants’ impressions regarding whether the scientist acts with the interests of others at heart and not just personal aims or benefit. This subscale could be considered similar to McCroskey and Teven’s [29] “Goodwill” dimension. However, unlike earlier concepts of goodwill, all items that make up benevolence in the METI can be applied for measurement even in situations in which the expert is not known personally to the recipient. Therefore, it allows us to measure laypeople’s benevolence assignments to scientists they encounter online, for example, on websites, social networks, or in science blogs. Developmental psychology has shown that children are sensitive to the intentions of a source of information, for example, the intention to help others [34,58]. In science communication, the benevolence of an expert is defined mostly through a scientist conducting research to benefit the greater good. The responsibility and ethicality of a scientific expert toward the general public might provide evidence of benevolence [59].

Empirically, the three-factor structure of the METI was first revealed by exploratory factor analysis (EFA) and then confirmed by confirmatory factor analysis (CFA) with a new and larger sample. To the best of our knowledge, no previously existing scale measuring trustworthiness or credibility has been tested in both these ways. The results suggest a good fit of the statistical model derived from EFA (Study 1) to the data of Study 2 tested with CFA. However, it should be noted that the subscale integrity did not reproduce perfectly in the CFA because communalities reached only .3 for two items in the subscale integrity. Thus, this dimension might comprise more than one aspect of integrity. It might be that the adherence to scientific standards entails not only the good character but also the appropriate values of an expert.

All in all, we were able to show empirically that judgments of epistemic trustworthiness are made up of three dimensions, namely expertise, integrity, and benevolence–a conclusion already suggested theoretically [42]. Furthermore, research on epistemic trust with children [2] along with earlier studies of source credibility [57], interpersonal trust [41], and trust within health and risk communication [39,55] have pointed toward epistemic trust consisting of subsidiary dimensions of similar content that can be ascribed to an informant. Hence, these dimensions make up the assessments laypeople undertake when they are challenged to decide whether they can defer to a certain source of knowledge or information about science, for example, a scientist or a website.

Furthermore, in one experimental study (study 3), it was shown that all three dimensions of the METI are sensitive to variation in source characteristics. In this study, participants were presented with six different descriptions of authors varying in expertise, integrity and benevolence. Not only did participants use the three dimensions of the METI in accordance with the dimensions targeted in the description, they also rated the authors most different (comparing low vs. high trustworthiness) on the dimension targeted. Even though the three factors seem to overlap to some extent, as a description of a highly trustworthy author on one dimension did lead to higher ratings on the other dimensions (integrity and benevolence especially showed this relation), the results show that the three factors were clearly distinguishable. If one dimension was presented to be low in the author description, this yielded the lowest rating on the respective subscale. If it was presented to be high, this led to highest ratings on the subscale. This was true for each subscale of the METI.

Further research should address how perceptions of epistemic trustworthiness would lead to actual epistemic trust. Actual epistemic trust would be indicated for example if a layperson would actually follow the advice of an expert or if she would turn to the expert for further information. In future studies it would be worthwhile to further assess in which ways trustworthiness evaluations on the respective scales of the METI predict epistemic trust.

In the three studies scientific information was introduced in blog entries written by authors who were indicated to be scientists or researchers (with one exception in study 3, in which a student was introduced as author in one condition). Blog entries were used, because they always have one distinctive source which is (mostly) easy to identify, while websites, journalistic articles or online dictionary entries may have multiple, sometimes indistinguishable sources. However, if the source to be rated is specified, we will suggest applicability of the METI for evaluation of this source.

We argue that the METI may go beyond inventories measuring general trust in scientific authority [60] or general trust in scientists belonging to a specific research field [59]. Unlike these inventories, METI does not measure the trust that is generally placed in experts. Instead, it is directed toward the epistemic trustworthiness assigned to one specific expert in a specific situation in which a layperson decides whether to place epistemic trust in that one expert and rely on the information that she or he is giving. The METI also shows that laypeople assign trustworthiness on three separate dimensions, namely expertise, integrity, and benevolence. Hence, the METI offers a way of measuring the processes involved in laypeople’s deference to experts in today’s digital knowledge society in more detail. Our society is characterized by highly specialized, and vastly distributed knowledge, which is nonetheless accessible within seconds via online search engines [61]. We argue that the ability to evaluate the epistemic trustworthiness of scientific experts will become an increasingly important competence, because (as has been argued before) scientific facts may defy firsthand experience [2], and, moreover, the public’s understanding of scientific issues is bounded [4]. Nonetheless, laypeople are challenged to make up their mind about everyday problems that entail scientific knowledge (e.g., “Did humans cause climate change?”), and they sometimes even decide to take action (e.g., “Should I purchase a hybrid car to counteract global warming?”). Evaluating the experts’ trustworthiness may be a way to cope with the science underlying such questions [4,7]. Hence, the METI offers insights into some of the processes of reasoning about scientific information.

The METI could be applicable to different contexts than science communication. We believe that trustworthiness judgments might well come into play in other situations involving communication. In interpersonal communication between interlocutors who do not yet know each other such as virtual teams or social media, judgments of epistemic trustworthiness are being made continuously. Future research could use METI to measure trustworthiness judgments within other domains.

## Supporting Information

S1 Appendix Study materials from studies 1 and 2. (DOCX)

S2 Appendix Study materials from Study 3. (DOCX)

S1 Table Means, skewness, and kurtosis for individual items (Study 2). (DOCX)

S2 Table Means, skewness, and kurtosis for all experimental conditions, and for the three scales (Study 3). (DOCX)
