# Supplementary material for: Measuring Laypeople’s Trust in Experts in a Digital Age: The Muenster Epistemic Trustworthiness Inventory (METI)
Source: PLoS One. 2015 Oct 16;10(10):e0139309. doi: 10.1371/journal.pone.0139309 (PMC4608577; doi:10.1371/journal.pone.0139309)
Supplement: S1 Appendix — (DOCX) [file pone.0139309.s001.docx]

## S1 Appendix. Study materials from studies 1 and 2

### Blog entry

Breakthrough in neuroenhancement!

[…] In recent years, I have been doing research on the drug Serofinil, which was originally used for treating migraine […]. In a small study, we investigated whether cognitive performance could be enhanced by the drug Serofinil. For this, we treated 25 students of medicine with a dose of 0.23 mg Serofinil for 4 weeks. Another 25 students of medicine did not receive any medication. In subsequent tests of cognitive performance (Brickenkamp, 1962; Jacobs, 2013), the group treated with Seronifil fared better than the group that did not receive neuroenhancing drugs. […] Thus, we can conclude that Serofinil is suitable for use while engaging in demanding cognitive tasks such as studying for an exam. […] Until today, no drug has shown effects this large in enhancing cognitive performance!

We aim to have this drug authorized by the *Bundesinstitut für Arzneimittel und Medizinprodukte* in 2014, so that […] people can gain from the positive effects of Serofinil. Nonetheless, more studies will have to be conducted before Serofinil can be approved for use by healthy people.
