# Supplementary material for: Measuring Laypeople’s Trust in Experts in a Digital Age: The Muenster Epistemic Trustworthiness Inventory (METI)
Source: PLoS One. 2015 Oct 16;10(10):e0139309. doi: 10.1371/journal.pone.0139309 (PMC4608577; doi:10.1371/journal.pone.0139309)
Supplement: S2 Appendix — (DOCX) [file pone.0139309.s002.docx]

## S2 Appendix. Study materials from Study 3

### Blog entry

Research News: When the brain doesn’t block distractions.

Smartphone alerts, traffic noise, a colleague speaks on the phone: We are constantly subject to distractions. I want to report a new and interesting study about this topic: concentration.

Researchers noticed that some patients suffering from migraines reported problems with concentration up to five hours before a migraine episode. Their concentration was limited to the extent that they could not focus on one task and were distracted by even small sensory input.

The study found that one nerve strand is crucial for the ability to concentrate. It proceeds between those areas of the brain, which first get sensory input and those which control the volley of input. This nerve strand was supplied with blood to a far lesser extent in migraine patients suffering from problems with concentration than in patients who did not report such symptoms.

On the long run, such findings could help patients suffering from migraine, if a drug could be devised that increases the supply with blood in this nerve strand. Also, people not suffering from migraine could hope to benefit from research like this. Who isn’t susceptible to distraction some days, wishing to stay focused?
