# Supplementary material for: Measuring Laypeople’s Trust in Experts in a Digital Age: The Muenster Epistemic Trustworthiness Inventory (METI)
Source: PLoS One. 2015 Oct 16;10(10):e0139309. doi: 10.1371/journal.pone.0139309 (PMC4608577; doi:10.1371/journal.pone.0139309)
Supplement: S1 Table — (DOCX) [file pone.0139309.s003.docx]

**S1 Table: Means, skewness, and kurtosis for individual items (Study 2).**

| **Item** | **Mean (SD)** | **Skewness** | **Kurtosis** |
| --- | --- | --- | --- |
| competent–incompetent | 4.02 (1.368) | .238 | -.674 |
| intelligent–unintelligent | 3.40 (1.361) | .541 | -.231 |
| well educated–poorly educated | 3.63 (1.341) | .292 | -.334 |
| professional–unprofessional | 4.19 (1.523) | .061 | -.887 |
| experienced–inexperienced | 3.91 (1.350) | .211 | -.438 |
| qualified–unqualified | 3.92 (1.404) | .245 | -.668 |
| sincere–insincere | 4.15 (1.444) | .079 | -.778 |
| honest–dishonest | 4.00 (1.434) | .171 | -.556 |
| just–unjust | 4.83 (1.347) | .057 | .253 |
| fair–unfair | 4.94 (1.392) | .236 | .095 |
| moral–immoral | 3.86 (1.189) | -.421 | -.385 |
| ethical–unethical | 4.86 (1.541) | -.347 | -.383 |
| responsible–irresponsible | 4.52 (1.308) | -.440 | -.499 |
| considerate–inconsiderate | 3.95 (1.212) | .024 | -.436 |
