# Supplementary material for: Measuring Laypeople’s Trust in Experts in a Digital Age: The Muenster Epistemic Trustworthiness Inventory (METI)
Source: PLoS One. 2015 Oct 16;10(10):e0139309. doi: 10.1371/journal.pone.0139309 (PMC4608577; doi:10.1371/journal.pone.0139309)
Supplement: S2 Table — (DOCX) [file pone.0139309.s004.docx]

**S2 Table: Means, skewness, and kurtosis for all experimental conditions, and for the three scales (Study 3)**

| **Condition** | **Dependent Variable** | **Mean (SD)** | **Skewness** | **Kurtosis** |
| --- | --- | --- | --- | --- |
| A1B1:  Low expertise | Expertise | 3.80 (.91) | 0,00 | -0,22 |
|  | Integrity | 4.62 (.84) | 0,01 | 0,04 |
|  | Benevolence | 4.45 (.82) | -0,26 | 1,64 |
| A1B2:  High expertise | Expertise | 6.37 (.88) | -2,42 | 7,40 |
|  | Integrity | 4.99 (1.01) | 0,36 | -0,77 |
|  | Benevolence | 5.01 (1.07) | 0,16 | -0,48 |
| A2B1:  Low integrity | Expertise | 4.75 (1.02) | -0,31 | -0,30 |
|  | Integrity | 2.40 (.91) | 0,55 | -0,10 |
|  | Benevolence | 2.50 (1.00) | 0,46 | -0,22 |
| A2B2:  High integrity | Expertise | 5.90 (.90) | -1,23 | 2,78 |
|  | Integrity | 5.93 (.98) | -0,94 | 0,88 |
|  | Benevolence | 5.79 (1.01) | -0,93 | 1,39 |
| A3B1:  Low benevolence | Expertise | 4.74 (1.08) | -0,24 | -0,71 |
|  | Integrity | 2.41 (1.04) | 0,74 | 0,77 |
|  | Benevolence | 1.88 (1.00) | 1,84 | 4,50 |
| A3B2:  High benevolence | Expertise | 5.91 (.88) | -0,84 | 0,83 |
|  | Integrity | 5.67 (.99) | -0,43 | -0,73 |
|  | Benevolence | 5.87 (1.02) | -0,98 | 0,82 |
